# Apple cider vinegar for prevention of urinary lithiasis (APUL): a randomized crossover trial

**DOI:** 10.1007/s11255-025-04944-x

**Published:** 2025-12-06

**Authors:** Benjamin Baker, Christine Herforth, Josiah Low, Ryan Craig, Matthew Christman

**Affiliations:** 1 Department of Urology, Naval Medical Center San Diego, 34800 Bob Wilson Dr, San Diego, CA 92134 USA; 2 Present Address: Department of Urology, University of California San Diego, 200 W. Arbor Dr, San Diego, CA 92103 USA; 3 Present Address: Department of Urology, University of Texas Southwestern Medical Center, 5323 Harry Hines Blvd, Dallas, TX 75390 USA; 4 Present Address: Cook Children’s Urology, 1500 Cooper St, Fort Worth, TX 76104 USA

**Keywords:** Urolithiasis, Kidney stone, Prevention, Apple cider vinegar, Complementary medicine

## Abstract

**Purpose:**

To evaluate the potential stone-preventative effects of apple cider vinegar (ACV) supplementation on 24-h urinary citrate, pH, and volume.

**Methods:**

We performed a randomized crossover trial testing ACV, coconut water, lemon water, and orange soda. Participants were non-stone formers randomized to two of the four beverages. Participants consumed one assigned beverage daily for one week, underwent a two-week washout period, and then consumed the second assigned beverage. Three 24-h urine collections were planned: one baseline collection prior to beverage consumption, one following completion of the first treatment week, and one following completion of the second week.

**Results:**

Twenty participants were enrolled. Eight were female. Six consumed ACV, six coconut water, eight lemon water, and six diet orange soda. ACV did not significantly change 24-h urinary parameters. Coconut water (+ 273.8 mg/24 h, 95% CI 67.9–480 mg/24 h) and lemon water (+ 167.7 mg/24 h, 95% CI 16–319 mg/24 h) were associated with increased urinary citrate. Effects on urine pH and volume were not significant in any treatment arms.

**Conclusion:**

ACV did not significantly change 24-h urine parameters. Coconut water and lemon water increased urinary citrate. Further study is necessary to validate these findings in a larger cohort. Registered with ClinicalTrials.gov (NCT04073719, 8/28/2019).

## Introduction

Urolithiasis is a common condition in the United States, affecting approximately 10% of men and 7% of women, with incidence continuing to rise [1–3]. Its associated costs are significant, with nearly 2 million yearly primary care visits and more than 600,000 emergency department visits attributable to urolithiasis incurring an estimated annual national expenditure of $2.1 billion [2]. Furthermore, stone formers have been shown to experience worse health-related quality of life across multiple domains [4].

To mitigate these burdens, stone prevention remains a cornerstone of urolithiasis management. Current literature supports dietary interventions such as increased hydration, restriction of dietary sodium, oxalate, non-dairy animal protein, and increased fruit and vegetable intake to reduce stone formation in stone formers. Although several pharmacologic agents have demonstrated efficacy in preventing stone formation, adherence to medical regimens remains a challenge [5]. Potassium citrate, for instance, is considered a relatively benign pharmacologic option for stone prevention, yet has reported long-term adherence rates as low as 42% [6].

An under-researched aspect of urolithiasis management is the role of complementary and alternative medicines (CAM), such as vitamins, herbs, and supplements. Patients are often exposed to and develop interests in CAM outside of the formal medical setting. One study of first-time and recurrent stone formers found that 50% of patients were taking some form of CAM. Eighty percent of these patients had heard of CAM, but the majority had received this information from a source other than their physician [7]. While CAM encompasses a massive range of products, those that are high in alkali citrate and total alkali content are of particular interest, as these components have been demonstrated to be anti-lithogenic to varying degrees [8–11]. For instance, there is evidence that orange juice and, to a lesser extent, lemon juice and coconut water may be protective against stone formation [12–16]. One popular CAM that has never been prospectively studied is apple cider vinegar (ACV). The only existing data on ACV is a chemical analysis, which showed an alkali load of 21 mEq/L, which could potentially increase citrate excretion through systemic alkalinization [17].

The purpose of this study was to serve as a physiologic, proof-of-concept evaluation of the effects of ACV, coconut water, diet orange soda, and lemon water on 24-h urine collection parameters in healthy volunteers. Given the relative tolerability and accessibility of these CAM beverages, any beneficial effects on urinary parameters could aid patient decision-making when choosing a CAM and support the design of larger prospective studies.

## Methods

This was a single-center, prospective, exploratory, randomized trial. A nested crossover design was utilized to increase the effective sample size per treatment arm. Neither patients nor members of the public were involved with the design or execution of the study. Patients seen in the Department of Urology at Naval Medical Center San Diego (NMCSD) between August 2019 and May 2021 without a diagnosis of urolithiasis were invited to participate. Exclusion criteria were a known diagnosis of chronic kidney disease (CKD), gastroesophageal reflux disease (GERD), current use of antihypertensive medications, and known pregnancy. Prospective participants also underwent a screening basic metabolic panel and were excluded if this was abnormal.

After consent was obtained, randomization was performed electronically using permuted blocks to two of four possible beverages: ACV (Bragg Live Food Products, LLC, Santa Barbara, CA), coconut water (Taste Nirvana Real Coconut Water, Taste Nirvana, Corona, CA), diet orange soda (Sunkist Diet Orange Soda, Keurig Dr. Pepper, Burlington, MA), or lemon water (ReaLemon, Keurig Dr. Pepper, Burlington, MA). Investigator CH had access to the allocation sequence. Blinding was not performed. All participants provided a baseline 24-h urine collection (UroRisk^®^, Quest Diagnostics, Secaucus, NJ). Participants were instructed to drink a daily serving of their first assigned beverage for seven days, after which they immediately completed a 24-h urine collection. This was followed by a two-week washout period during which participants were directed to avoid consumption of any of the study beverages. This duration was deemed adequate based on existing literature on the approximate four-hour washout time of an acute dietary citrate load [18]. Finally, participants were instructed to drink a daily serving of their second assigned beverage for seven days, followed by a final 24-h urine collection. Participants were instructed to document any side effects experienced with the consumption of their assigned beverage.

ACV was prepared by diluting 30 mL of bottled ACV with water to a total volume of 1L. Lemon water was prepared by diluting 120 mL of lemon concentrate with water to a total volume of 1L. These ratios were determined with the assistance of a registered dietician to ensure safety and palatability. A zero-calorie sweetener (Splenda^®^, Heartland Consumer Products, Carmel, Indiana) was allowed to be added to both to taste. Coconut water and diet orange soda were consumed as packaged. Diet orange soda included aspartame and acesulfame potassium as sweetener agents. Approximately 1 L of total volume was consumed daily for each beverage (Table 1). Characteristics of treatment beverages were recorded, with citrate and alkali loads calculated based on values in existing literature [7, 16, 19, 20]. No data was found on the total alkali load of lemon concentrate.

**Table 1:** Baseline characteristics by treatment arm

	Overall	ACV	Coconut water	Lemon water	Diet orange soda	*p*-value
Age (median [IQR])	31 [24.5–34]	32.5 [31–34.8]	30 [27.3–33.5]	33.5 [27.8–35]	32 [31–33.8]	0.9514
BMI	23.3 [21.7–25.1]	25.1 [23.7–26.3]	22.9 [22.2–23.5]	23.4 [22.6–25.6]	21.7 [20.6–23]	0.3757
*Sex (count)*						
F	8	3	2	4	3	0.902
M	7	3	4	4	3	
Volume (daily, mL)	na	1000	960	1000	1065	
Alkali citrate load (daily, mmol)	na	0	2.02	21.3	3	
Alkali total load (daily, mEq)	na	21	13.25	–	11.2	

Basic demographic information and a basic metabolic panel were collected on all participants. The primary outcome was adjusted mean change from baseline in 24-h urine citrate after each seven-day treatment period. Secondary outcomes were adjusted mean change from baseline in 24-h urine pH and volume after each treatment period. As the study was designed as an exploratory analysis, we deferred formal power calculation and relied on the conditional power of our analysis once enrollment was closed. Analysis of variance and Fisher’s exact test were used to compare baseline age, BMI, and sex between treatment arms. Linear mixed-effects models were fitted using assigned beverage, period, sequence, and baseline urine parameters as fixed effects and subjects as a random effect, with adjusted mean change of parameter from baseline as the outcome. All patients who completed at least one arm were included in the analysis. All analysis was performed in R (R Core Team, Vienna, Austria) [21]. This study was approved by the NMCSD Institutional Review Board under protocol number NMCSD.2019.0026 and was registered on ClinicalTrials.gov under NCT04073719.

## Results

**Fig. 1:**
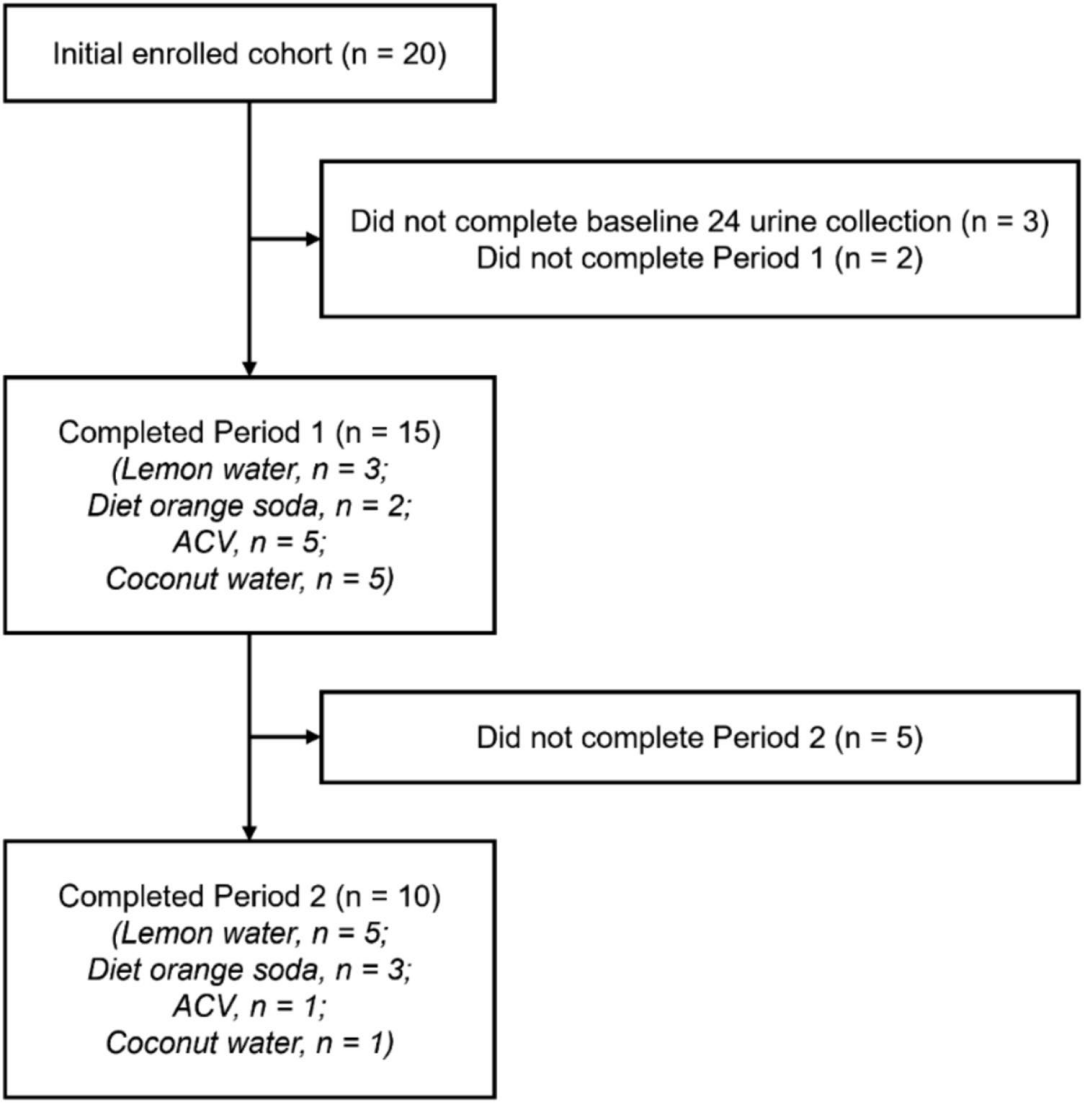
CONSORT diagram

After initial screening, 20 participants were enrolled, eight of whom were female. Median age was 31 (IQR 26–34) and median BMI was 23.3 (IQR 21.9–25.2). Treatment arms did not differ significantly in the distribution of age, BMI, or sex (Table 1). Fifteen participants completed Period 1. Ten of those participants proceeded to complete Period 2. Total effective sample size of 25. In total, six participants completed treatment with ACV, six with coconut water, eight with lemon water, and six with diet orange soda (Fig. 1). Patient accrual was stopped prior to reaching recruitment goals due to changes in department personnel and loss of research staff.

**Fig. 2:**
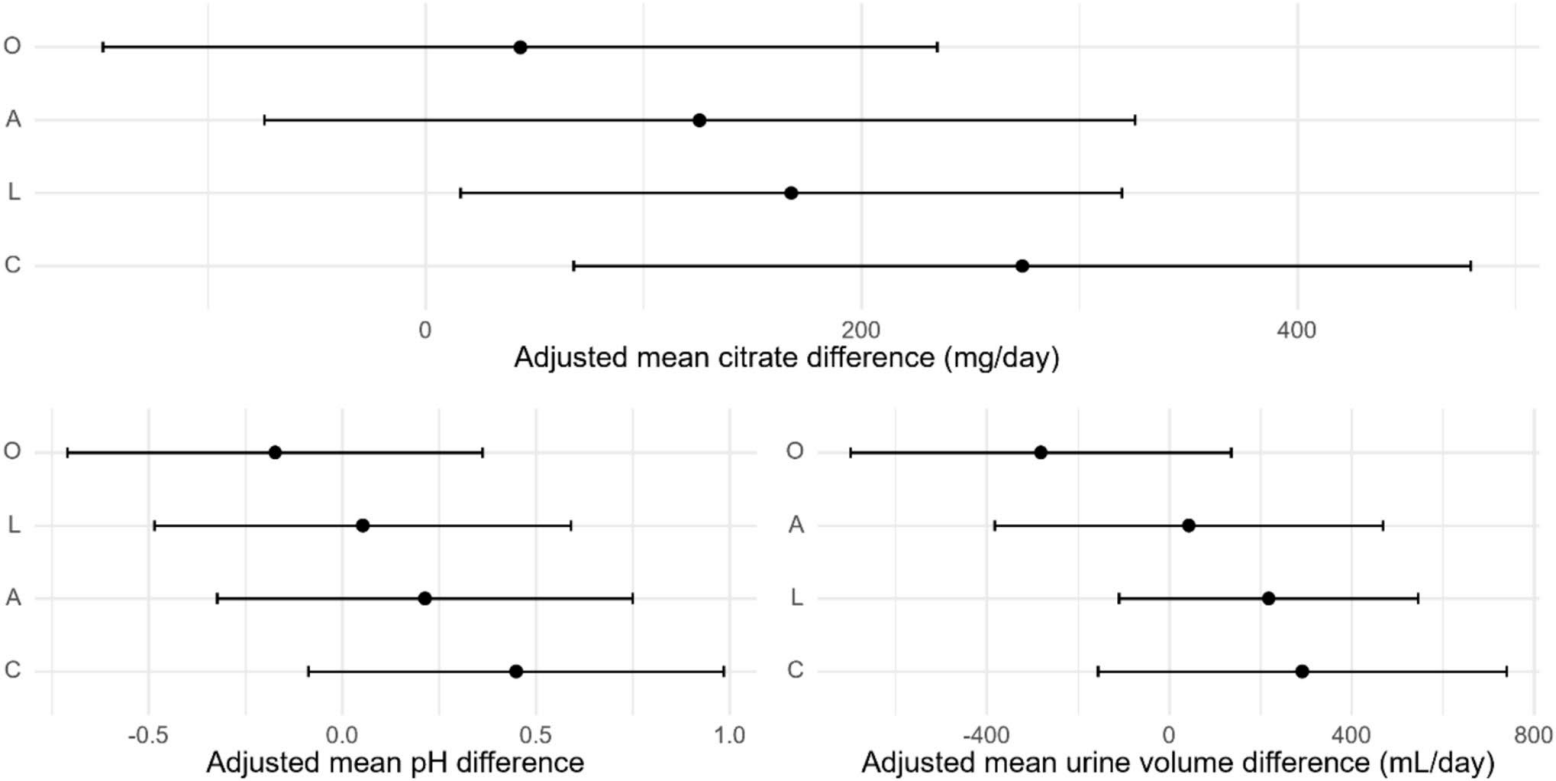
Adjusted mean difference by treatment. Bars represent a 95% confidence interval. O – diet orange soda; A – apple cider vinegar; L – lemon water; C – coconut water

Baseline and post-treatment 24-h urinary citrate, pH, and volume were measured (Table 2). A significant adjusted mean change in 24-h urinary citrate was seen with consumption of coconut water (+ 273.8 mg/24 h, 95% CI 67.9–480 mg/24 h) and lemon water (+ 167.7 mg/24 h, 95% CI 16–319 mg/24 h). Adjusted mean change of citrate for ACV and diet orange soda, as well as adjusted mean change of pH and volume for all treatments, was noted to have 95% confidence intervals that crossed zero and were not considered to be significant (Fig. 2; Table 3). No adverse effects were reported.

**Table 2:** Urinary parameters by treatment arm (baseline and post-treatment)

	ACV		Coconut water		Lemon water		Diet orange soda	
	Baseline (median [IQR])	Post-treatment (median [IQR])	Baseline	Post-treatment	Baseline	Post-treatment	Baseline	Post-treatment
Citrate (mg/24 h)	303.5 [255–336.3]	253.5 [230–441.3]	367 [245.3–419]	543.5 [445–671.3]	317 [226.3–407]	244 [192.5–444.8]	316 [216–576]	540 [320–759]
Volume (mL)	1550 [1337.5–1725]	1667.5 [1433.8–2175]	1325 [1025–1775]	1412.5 [1175–2043.8]	1525 [1167.5–1787.5]	1520 [1281.3–1806.3]	1600 [1250–1650]	1500 [1400–2330]
pH	6.3 [6–6.7]	6.2 [6–6.4]	6.1 [5.9–6.4]	6.5 [6.1–6.8]	6.1 [5.7–6.8]	6.1 [5.8–6.5]	6 [6–6.5]	6.1 [5.7–6.6]

**Table 3:** Linear mixed-effect model results

	ACV		Coconut water		Lemon water		Diet orange soda	
	Adjusted mean change [95% CI]	Standard error	Adjusted mean change	Standard error	Adjusted mean change	Standard error	Adjusted mean change	Standard error
Citrate (mg/24 h)	+ 125.7 [− 74.1–325]	88.9	+ 273.8 [67.9–480]	89.9	+ 167.7 [16–319]	67.9	+ 43.4 [− 148–235]	83.8
Volume (mL)	+ 42.6 [− 383–468]	189.00	+ 291 [− 157–739]	195.00	+ 218 [− 110–546]	146.00	− 282 [− 699–136]	182.00
pH	+ 0.21 [− 0.32–0.75]	0.21	+ 0.45 [− 0.088–0.99]	0.21	+ 0.53 [− 0.48–0.59]	0.21	− 0.17 [− 0.71–0.36]	0.21

## Discussion

In this randomized crossover study, one week of ACV consumption did not produce significant changes in 24-h urinary parameters. Coconut water and lemon water, however, significantly increased urinary citrate. This is consistent with existing literature demonstrating a similar effect in both prospective and retrospective studies [12, 13, 16]. Patel and colleagues previously showed that four days of coconut water consumption were associated with a 161 mg/day increase in urinary citrate. They noted that, despite the relatively low alkali citrate level in the beverage itself, the overall alkali load was quite high, leading them to hypothesize that coconut water might increase urinary citrate excretion mostly through systemic alkalinization resulting in secondary renal citrate excretion, as opposed to direct gastrointestinal absorption of alkali citrate [16]. This is presumably the same mechanism by which diet orange soda produced increased citrate and pH, as it similarly had a low alkali citrate level but a higher total alkali load.

A strength of our study lies in its randomized crossover design, which represents a first-of-its-kind study design in the urolithiasis literature and allows for an enhanced effective sample size for a given number of participants. Despite the absence of significant effects on urinary parameters, the novelty of studying ACV is also a strength of this work. Our selection of beverages was based on their known alkali citrate and total alkali loads. Of the beverages studied, coconut water appears to have the most convincing signal of an effect on urinary citrate. We acknowledge that our study failed to meet target accrual by a substantial margin. Our findings should therefore be interpreted as exploratory in nature, with the knowledge that the potential true effect of these beverages on urinary parameters may not have been detected due to significant underpowering.

Another limitation of our findings is the absence of adherence and diet recall data, which would have facilitated a per-protocol analysis and offered insight into the palatability and practicality of beverage consumption. There remains a need for further prospective study of both consumer beverages as well as other forms of CAM with adequate powering and enhanced capture of adherence data. Furthermore, blinding was impractical due to the inherent need for subjects to taste the treatment beverage. Attempts were made to mitigate this limitation by standardizing counseling of all participants prior to randomization. Lastly, our study was designed to assess the effect of the beverages of interest on healthy volunteers. Repeating this study in hypocitraturic stone formers would bolster generalizability to patients in real-world clinical practice.

## Conclusions

ACV did not demonstrate significant effects on 24-h urine parameters. Importantly, our study was underpowered to detect these potential associations based on a priori sample size calculations. Coconut water and lemon water were, by contrast, associated with significant increases in urinary citrate. Further study of these beverages may yield clinically relevant findings in a larger cohort.

## Data Availability

The deidentified dataset used for this study is available for review at 10.7910/DVN/ZEZPJF.
